# Combinational therapy with antibiotics and antibiotic-loaded adipose-derived stem cells reduce abscess formation in implant-related infection in rats

**DOI:** 10.1038/s41598-020-68184-y

**Published:** 2020-07-07

**Authors:** Junya Yoshitani, Tamon Kabata, Hiroshi Arakawa, Yukio Kato, Takayuki Nojima, Katsuhiro Hayashi, Masaharu Tokoro, Naotoshi Sugimoto, Yoshitomo Kajino, Daisuke Inoue, Ken Ueoka, Yuki Yamamuro, Hiroyuki Tsuchiya

**Affiliations:** 1 Department of Orthopaedic Surgery, Graduate School of Medical Sciences, Kanazawa University, 13-1 Takaramachi, Kanazawa, Ishikawa 920-8641 Japan; 2 Faculty of Pharmacy, Institute of Medical, Pharmaceutical and Health Sciences, Kanazawa University, Kanazawa, Japan; 3 Department of Pathology and Laboratory Medicine, Kanazawa University, Kanazawa, Japan; 4 Department of Parasitology, Graduate School of Medical Sciences, Kanazawa University, Kanazawa, Japan; 5 Department of Physiology, Graduate School of Medical Science, Kanazawa University, Kanazawa, Japan

**Keywords:** Drug discovery, Stem cells, Materials science

## Abstract

Implant-related infection is difficult to treat without extended antibiotic courses. However, the long-term use of antibiotics has led to the development of multidrug- and methicillin-resistant *Staphylococcus* *aureus.* Thus, alternatives to conventional antibiotic therapy are needed. Recently, mesenchymal stem cells have been shown to have antimicrobial properties. This study aimed to evaluate the antimicrobial activity and therapeutic effect of local treatment with antibiotic-loaded adipose-derived stem cells (ADSCs) plus an antibiotic in a rat implant-associated infection model. Liquid chromatography/tandem mass spectrometry revealed that ADSCs cultured in the presence of ciprofloxacin for 24 h showed time-dependent antibiotic loading. Next, we studied the therapeutic effects of ADSCs and ciprofloxacin alone or in combination in an implant-related infection rat model. The therapeutic effects of ADSCs plus antibiotics, antibiotics, and ADSCs were compared with no treatment as a control. Rats treated with ADSCs plus ciprofloxacin had the lowest modified osteomyelitis scores, abscess formation, and bacterial burden on the implant among all groups (*P* < 0.05). Thus, local treatment with ADSCs plus an antibiotic has an antimicrobial effect in implant-related infection and decrease abscess formation. Thus, our findings indicate that local administration of ADSCs with antibiotics represents a novel treatment strategy for implant-associated osteomyelitis.

## Introduction

Periprosthetic infections are a tremendous burden to patients and healthcare institutions worldwide^1^. With the increase in arthroplasty procedures and the ongoing development of drug-resistant microorganisms, the incidence of such infections has been increasing^1^. To address this challenge, novel treatments are necessary^1^.

*Staphylococcus* *aureus* (*S.* *aureus*) is one of the primary pathogens responsible for implant-associated osteomyelitis^2^. The ability of *S.* *aureus* to establish chronic, implant-associated infections and our inability to cure them are directly associated with its capacity to form biofilms, creating an environment where the bacteria can grow and persist while being protected from the patient’s immune response and antibiotics^3^. At present, systemic administration of antibiotics is the standard therapy for implant-associated infections. However, the long-term use of antibiotics has led to the development of multidrug-resistant and methicillin-resistant *S.* *aureus*^4^. Strategies for local antibiotic delivery to increase the antimicrobial concentration at the site of infection while keeping systemic levels low to avoid potential side effects have been investigated for several decades^5^. However, there still is an unmet need for alternatives to conventional antibiotic therapy for the management of chronic infections^4^.

Recently, mesenchymal stem cells (MSCs) have been shown to have antimicrobial properties^6–10^. MSCs reportedly participate in the innate immune response through the secretion of antimicrobial peptides^7^. Bone marrow-derived stem cells (BMSCs) can be loaded with antibiotics and other drugs, and MSCs including adipose-derived stem cells (ADSCs) co-administered with antibiotic therapy may be a novel effective, antimicrobial approach to the treatment of chronic, drug-resistant infections^5,11,12^. Among the various types of MSCs, ADSCs have numerous unique advantages. They are abundant in subcutaneous adipose tissues and can be easily harvested using a syringe or by minimally invasive lipoaspiration^13^. In addition, they contribute to the complex wound-repair processes, comprising inflammation, granulation, and remodelling^14,15^. While ADSCs are known to exert antibacterial activity, their activity in implant-related osteomyelitis has not been previously investigated. We hypothesized that ADSCs loaded with an antibiotic can exert an antimicrobial therapeutic effect in implant-related osteomyelitis. Therefore, we studied the effects of local treatment with ADSCs and ADSCs plus an antibiotic in a rat model of implant-associated osteomyelitis to evaluate their effectiveness in implant-related infection.

## Results

### Effect of the antibiotic on ADSCs

**Figure 1:**
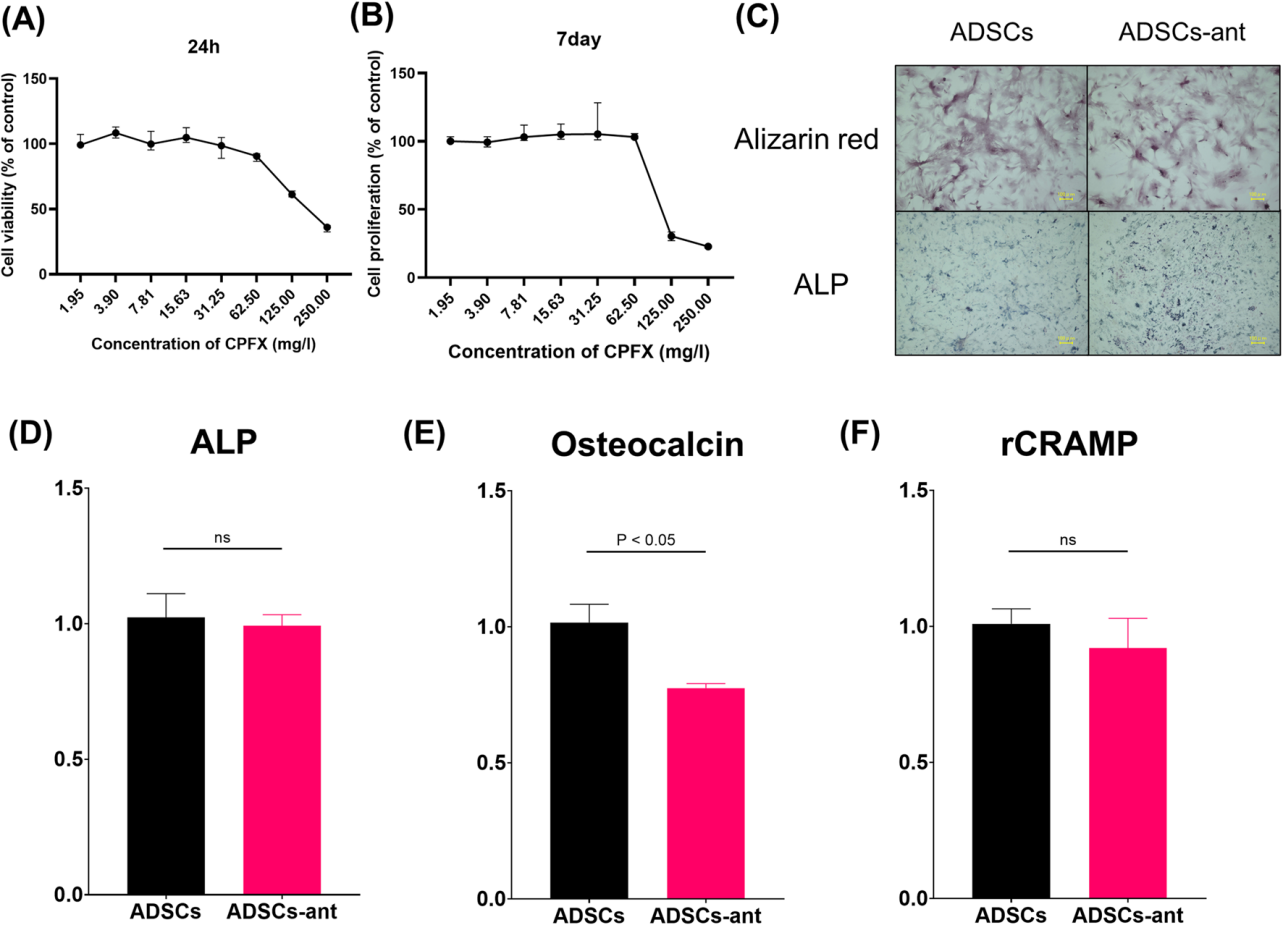
Cell viability based on the measurement of mitochondrial oxidative activity after exposure CPFX for 24 h (**A**) or 7 days (**B**). Bars represent the median ± interquartile range of the percentages of the control (100%). (**C**) Alizarin red and ALP staining of ADSCs and ADSCs loaded with 100 mg/L CPFX (ADSCs-ant) at 2 weeks. (**D**–**F**) RT-qPCR results. ALP, alkaline leukocyte phosphatase (**D**), osteocalcin (**E**), rCRAMP, rat cathelicidin-related antimicrobial peptide (**F**). There was a significant difference in osteocalcin, whereas there were no significant differences in ALP and rCRAMP between ADSCs and ADSCs-ant.

Ciprofloxacin (CPFX) dose-dependently suppressed the proliferation of ADSCs, with half-maximum inhibitory concentration (IC_50_) values of 99.5 mg/L at 24 h and of 103.6 mg/L at 7 days (Fig. 1A, B). Based on the findings, we established 100 mg/L as an optimal CPFX dose for priming ADSCs. Alizarin red and alkaline phosphatase (ALP) staining showed that ADSCs and antibiotic-loaded ADSCs were well differentiated at 2 weeks after osteogenic induction (Fig. 1C). Moreover, mRNA levels of *ALP* and osteocalcin were examined to determine the osteogenic capacity. Quantitative reverse-transcription (RT-q)PCR showed that antibiotic-loaded ADSCs had similar *ALP* mRNA levels, but reduced osteocalcin mRNA levels when compared to ADSCs (Fig. 1D, E). The mRNA level of rat cathelicidin-related antimicrobial peptide (*rCRAMP*) was also examined to assess the antimicrobial peptide secretion ability. *rCRAMP* was expressed to similar levels in both antibiotic-loaded ADSCs and ADSCs (Fig. 1F).

### Quantification of CPFX in and released from ADSCs

**Figure 2:**
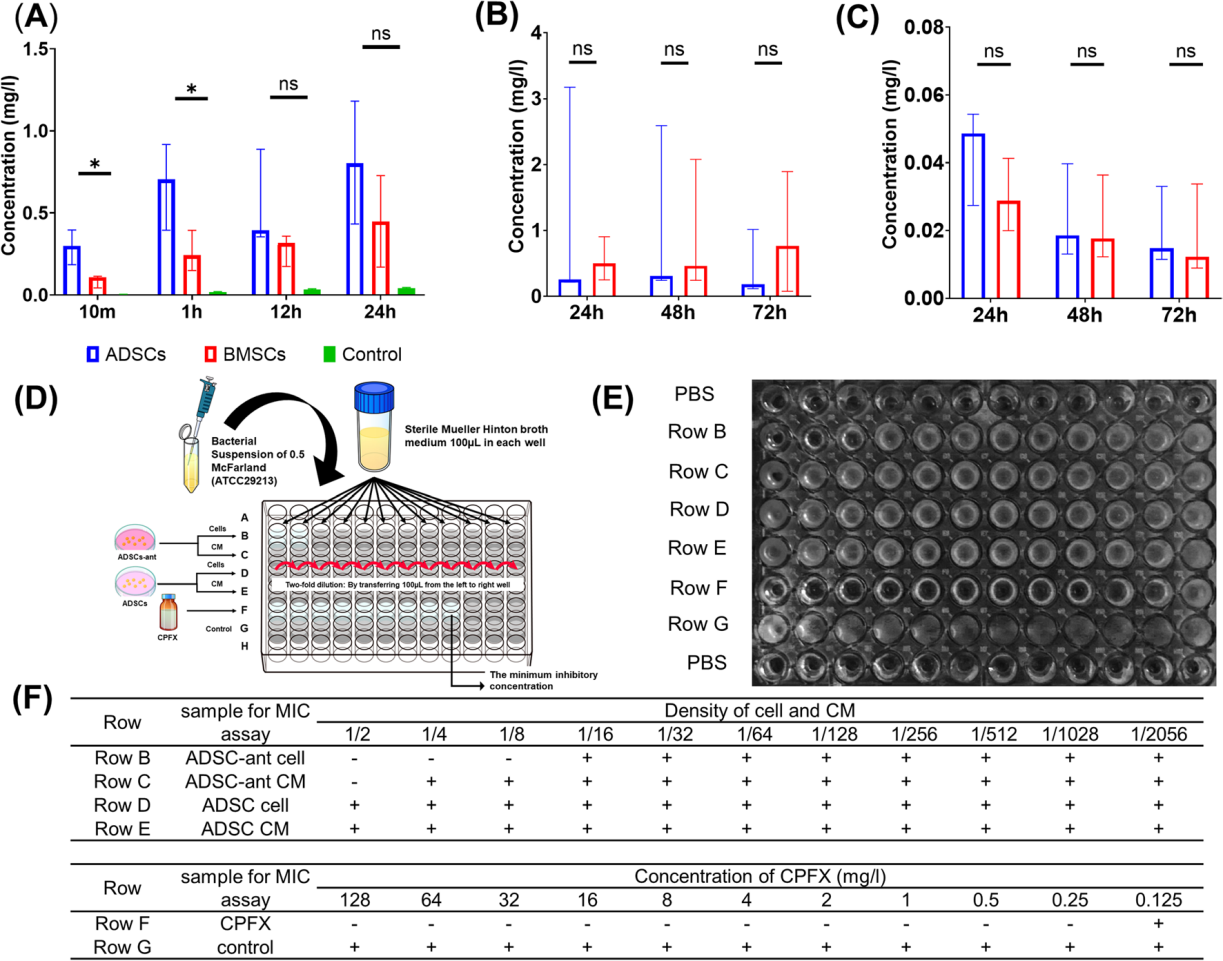
Evaluating the antibiotic loading and releasing ability of ADSCs. (**A**–**C**) CPFX concentrations in cells or CM determined by LC–MS/MS at the indicated time points after CPFX loading or release. (**A**) CPFX concentrations after loading onto ADSCs and BMSCs. *P < 0.05; ns, no significant difference. The green bar represents a control without cells to confirm that CPFX did not adsorb to the plate. (**B**) CPFX concentrations released by cells in CM. (**C**) CPFX concentrations in cells after release. The broth dilution method was used to evaluate the antimicrobial activity of ADSCs-ant and CM on *S.* *aureus*. The protocol is shown in (**D**), the results in (**E**). Row B: serial 1:2 dilution of ADSCs-ant, Row C: serial 1:2 dilution of CM of ADSCs-ant, Row D: serial 1:2 dilution of ADSCs, Row E: serial 1:2 dilution of CM of ADSCs, Row F: serial 1:2 dilution of CPFX (starting stock solution 128 mg/l; MIC at 1:2 dilution = 0.125 mg/l), Row G: control bacterial growth in medium without CPFX. (**F**) ADSCs-ant induced visible growth inhibition at a dilution of 1:8 (row B), and the CM of ADSCs-ant induced growth inhibition at a dilution of 1:2 (row C). Growth inhibition was not induced by ADSCs and their CM (rows D and E).

Both ADSCs and BMSCs showed a time-dependent loading of CPFX during 24 h of culture in the presence of CPFX (Fig. 2A). The concentration of CPFX in ADSCs was significantly higher than that in BMSCs (Fig. 2A). At 10 min and 1 h, CPFX concentrations significantly differed between ADSCs than BMSCs, whereas at 12 h and 24 h, no significant differences were detected (Fig. 2A). CPFX adsorption to the plate was hardly observed (Fig. 2A). In the release phase, CPFX was detected at 72 h in conditioned media (CM) of both ADSCs and BMSCs (Fig. 2B). The concentrations of CPFX in CM of ADSCs and BMSCs showed no significant differences at 24 h, 48 h, and 72 h after CPFX release (Fig. 2C). The antimicrobial activity of antibiotic-loaded ADSCs and CM of these cells was evaluated using the broth dilution method (Fig. 2D, E). The minimum inhibitory concentration (MIC) values for *S.* *aureus* were determined using a 1:2 serial dilution of a standard preparation of CPFX. Complete growth inhibition of *S.* *aureus* was observed at 0.125 mg/L of CPFX (Fig. 2F, row A). Antibiotic-loaded ADSCs induced visible growth inhibition at a dilution of 1:8 (Fig. 2F, row B), and the CM of the antibiotic-loaded ADSCs induced growth inhibition at a dilution of 1:2 (Fig. 2F, row C). Growth inhibition was not detected in ADSCs and CM of these cells (Fig. 2F, rows D and E). These results showed that ADSCs could load enough CPFX to exert antimicrobial activity against *S.* *aureus*.

### In-vivo analysis of implant-related infection in rats

**Figure 3:**
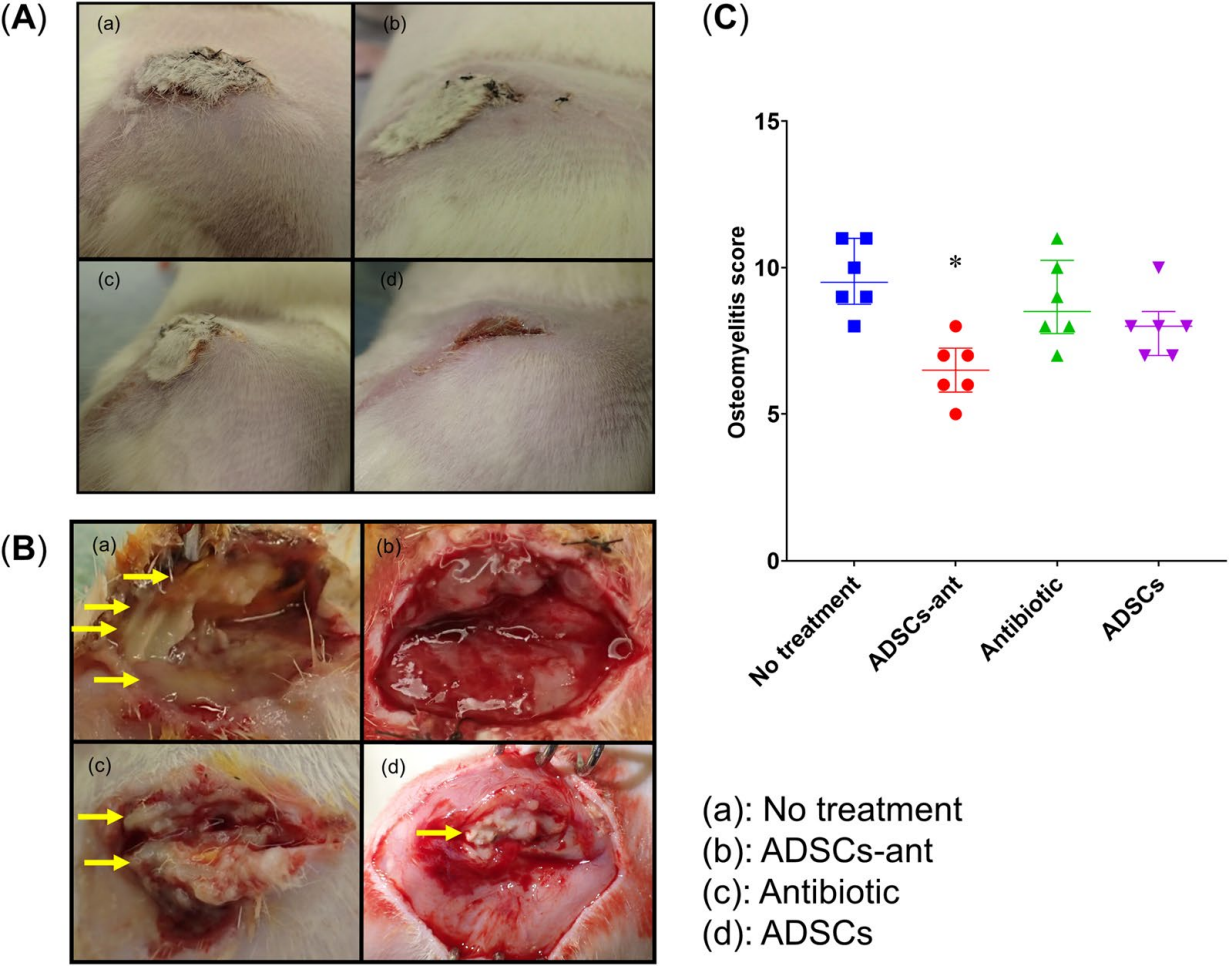
Evaluation of the modified osteomyelitis score. (**A**) No treatment, (**B**) ADSCs-ant, (**C**) antibiotic, (**D**) ADSCs. (**A**) Soft tissue swelling. (**B**) Abscess formation. The yellow arrow indicates abscess formation. (**C**) The ADSCs-ant group showed the lowest modified osteomyelitis score among all groups. *P < 0.05 by Kruskal–Wallis test followed by Dunn’s post-hoc test.

**Figure 4:**
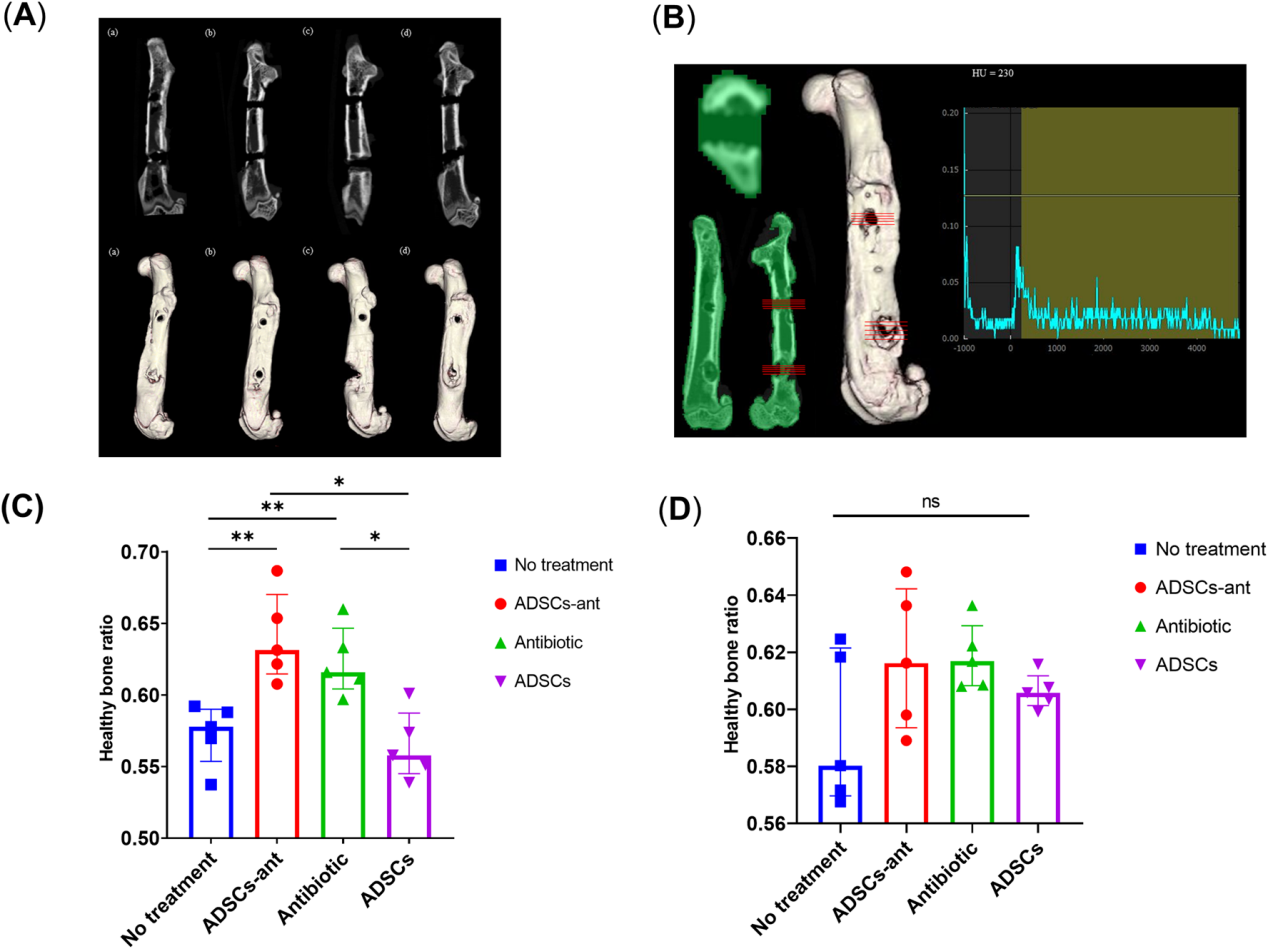
μCT imaging analysis of the femur of rats (LaTheta). (**A**) μCT image of a representative femur of each group; (1) no treatment, (2) ADSCs-ant, (3) antibiotic, (4) ADSCs. (**B**) Evaluation of the Hounsfield Unit value using the DICOM viewer software (Synapse Vincent). (**C**) Healthy bone ratio at the proximal screw in all groups. *P < 0.05 versus no treatment and ADSCs; **P < 0.001 versus no treatment and ADSCs; ns, no significant difference by Kruskal–Wallis test followed by Dunn’s post-hoc test. No treatment: mean 0.57, SD 0.07, range 0.47–0.72; ADSCs-ant: mean 0.64, SD 0.05, range 0.52–0.73; antibiotic: mean 0.62, SD 0.06, range 0.53–0.74; ADSCs: mean 0.56, SD 0.06, range 0.46–0.66. (**D**) Healthy bone ratio at the distal screw in all groups. There were no significant differences between groups by Kruskal–Wallis test followed by Dunn’s post-hoc test.

For *S.* *aureus* exposure, we coated the screw with 5 × 10^7^ colony-forming units (CFU), which induced infection in 100% of non-treated rats by day 7 day after surgery. Intra-rater reliability of the modified osteomyelitis score was assessed, and the intra-class coefficient was 0.902 (95% confidence interval, 0.850–0.947). The no-treatment group showed obvious swelling at the surgical site, whereas rats in the antibiotic-loaded ADSCs plus CPFX (ADSCs-ant) and ADSCs groups showed very limited swelling (Fig. 3A). Rats in the no-treatment group clearly showed abscess formation. Rats in the ADSCs-ant group showed very limited abscess formation, whereas those in the antibiotic and ADSCs groups showed moderate abscess formation (Fig. 3B). The no-treatment group had the highest modified osteomyelitis score, whereas the ADSCs-ant group had the lowest score among all groups (Kruskal–Wallis test followed by Dunn’s post-hoc test, *P* < 0.05; Fig. 3C). The antibiotic and ADSCs groups showed no significant differences when compared to the no-treatment group. Micro-computed tomography (μCT) analysis showed obvious osteolysis around the screw hole in the no-treatment, antibiotic, and ADSCs groups, especially around the distal screw hole (Fig. 4A). The healthy bone ratio of the proximal screws was significantly higher in the ADSCs-ant and antibiotic groups, and osteolysis around screw holes was significantly reduced in these groups when compared to the no-treatment and ADSCs groups (Fig. 4B, C). There were no significant differences in osteolysis of the distal screw holes among the groups (Fig. 4D).

### Histological assessment of abscess formation

**Figure 5:**
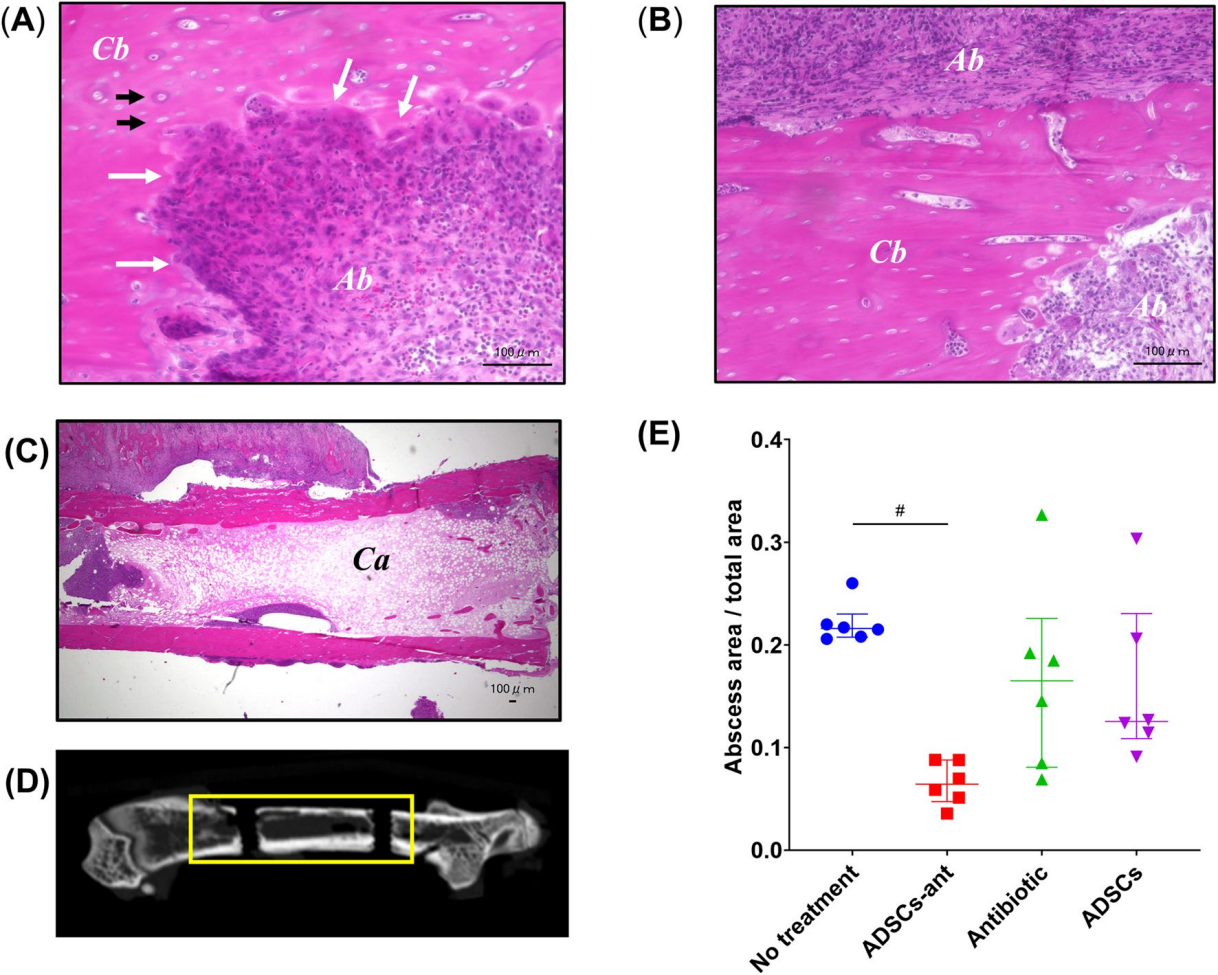
Histological analysis. (**A**–**C**) Microscopic images of formalin-fixed, H&E-stained paraffin sections. *Ab* indicates abscess formation. *Cb* indicates cortical bone. The white arrow indicates partial disappearance of the cortical bone. The black arrow indicates necrosis of the cortical bone. *Ca* indicates cancellous bone. (**D**) The abscessed area in total area was evaluated in three regions, including at the distal screw hall, the proximal screw hall, and the region between both screw halls. (**E**) Abscessed area in total area in all groups. The abscessed area was significantly lower in the ADSCs-ant group than in the no-treatment group (*P* < 0.05 by ordinary one-way ANOVA followed by Sidak’s post-hoc test).

Histological analysis provided apparent evidence of advanced spongy alteration, partial disappearance of cortical substance, and abscess formation (Fig. 5A–C). The abscessed area in the total area was evaluated in all groups (Fig. 5D). The abscessed area was significantly reduced in the ADSCs-ant group when compared with the no-treatment group (ordinary one-way ANOVA followed by Sidak’s post-hoc test, *P* < 0.05, Fig. 5E), whereas the antibiotic and ADSCs groups did not show reduced abscess formation. Rats in the ADSCs-ant group showed no abscess formation in the proximal screw hole. Rats in the no-treatment, antibiotic, and ADSCs groups showed abscess formation around the screw hole and the plate. Rats in the no-treatment and antibiotic groups showed necrotic cancellous bone in the central area, whereas animals in the ADSCs-ant and ADSCs groups had viable cancellous bone in this area. All groups showed abscess formation in the distal screw hole, although ADSCs-ant-treated rats had smaller abscessed areas than others (Supplementary Fig. S1).

### Effects of ADSCs-ant on implant and soft-tissue bacterial burden

**Figure 6:**
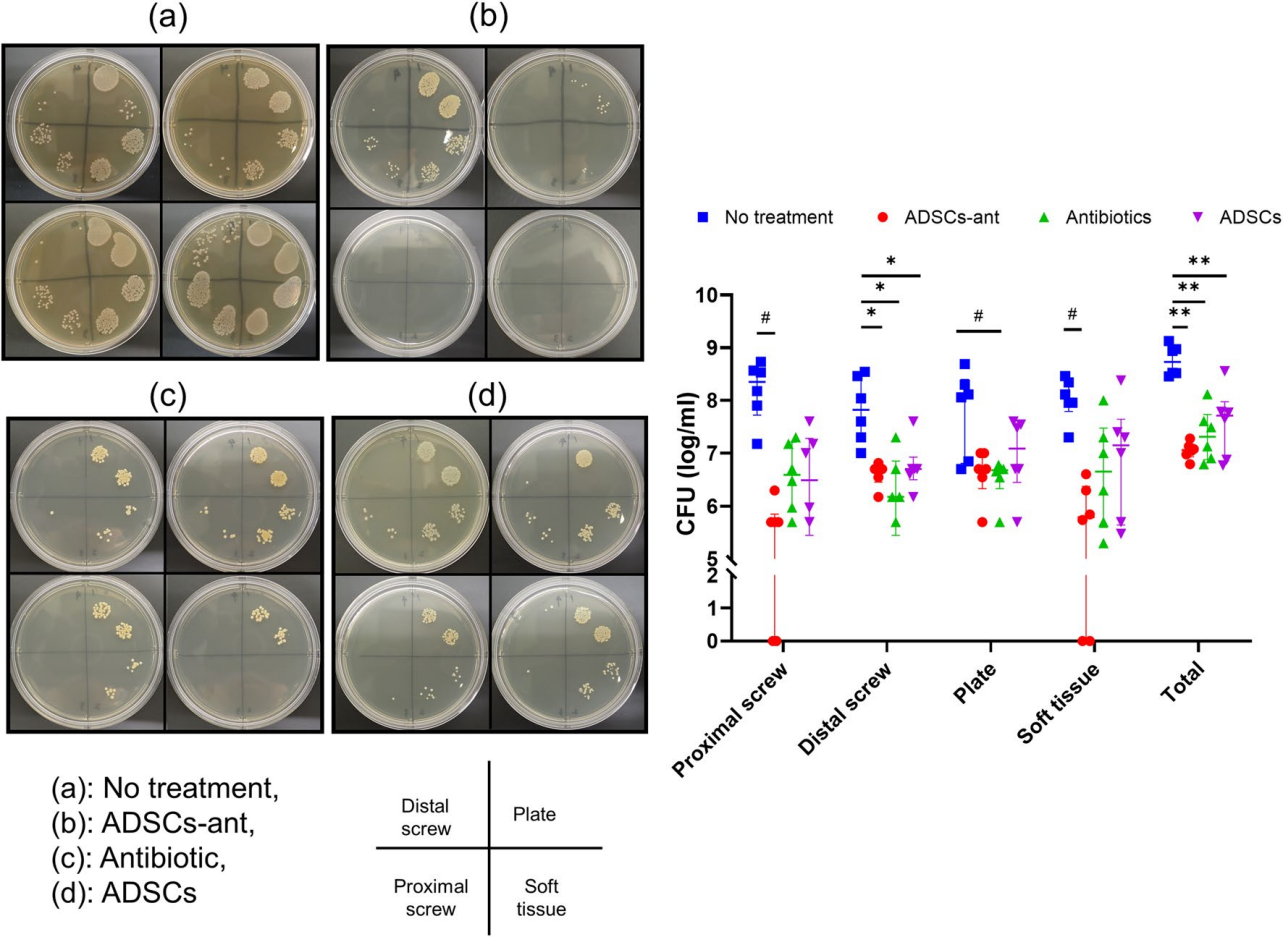
Effects of ADSCs and antibiotic on bacterial burden on the implant and in soft tissue as determined by CFU analysis. (**a**) No treatment, (**b**) ADSCs-ant, (**c**) antibiotic, (**d**) ADSCs. #*P* < 0.05, by Kruskal–Wallis test followed by Dunn’s post-hoc test; *P < 0.05, by ordinary one-way ANOVA followed by Sidak’s post-hoc test; **P < 0.05 by Brown-Forsythe and Welch ANOVA followed by Dunnett’s T3 post-hoc test.

Bacterial infection was detected in all rats in the no-treatment group. Treatment with ADSCs-ant significantly suppressed the bacterial burden on the proximal screw compared to that in no treatment (Kruskal–Wallis test followed by Dunn’s post-hoc test, *P* < 0.001, Fig. 6). ADSCs-ant, antibiotic, and ADSCs significantly reduced the bacterial burden on the distal screw compared to no treatment (one-way ANOVA followed by Sidak’s post-hoc test, *P* < 0.05, *P* < 0.001, *P* < 0.05, respectively). Only the antibiotic group showed significantly decreased bacterial burden on the plate when compared to the no-treatment group (Kruskal–Wallis test followed by Dunn’s post-hoc test, *P* < 0.05). ADSCs-ant induced a significant decrease in bacterial burden in the soft tissue as compared to that in no treatment (Kruskal–Wallis test followed by Dunn’s post-hoc test, *P* < 0.05). ADSCs-ant, antibiotic, and ADSCs significantly reduced total bacterial burden compared to that in no treatment (Brown-Forsythe and Welch ANOVA followed by Dunnett’s T3 post-hoc test, *P* < 0.0001, *P* < 0.05, and *P* < 0.05, respectively).

## Discussion

Our in vitro results demonstrated that ADSCs could be primed with CPFX by a simple method, and the concentration of CPFX in the ADSCs increased with exposure time. The in vivo studies demonstrated that ADSCs combined with CPFX improved osteomyelitis scores and decreased osteolysis and bacterial loads in rats with implant-related infection as compared to that in no treatment in a rat model of implant-related infection. Furthermore, only the ADSCs-ant group showed a significant decrease in abscess formation among all groups. ADSCs alone lowered bacterial loads compared to no treatment, but the effect was not larger than that of antibiotic alone. These results demonstrated that ADSCs have an antimicrobial effect and effectively decrease the bacterial burden on the implant, and the effect was enhanced when the ADSCs were combined with an antibiotic.

Owing to their differentiation plasticity, immunomodulatory properties, angiogenic modulation, and paracrine support^16–19^ , MSCs have been investigated in a wide spectrum of diseases, as evidenced by the approximately 500 trials registered in the ClinicalTrials.gov database of the National Institutes of Health (https://www.clinicaltrials.gov/, queried in December 2016)^20^. However, the use of local injection of ADSCs loaded and combined with an antibiotic to treat implant-related osteomyelitis had not been reported to date. Multiple, complementary mechanisms of action (both direct and indirect) likely account for the ability of MSCs to help control infections, although it is not fully understood whether the main weapon is the cell itself or its secretome^7,9^. They might act indirectly through their role in the host immune response against pathogens, especially in the dynamic coordination of pro- and anti-inflammatory elements of the immune system^21–23^ or by increasing the activity of phagocytes^24–26^. They might act directly through the secretion of antimicrobial peptides and proteins^27–30^ and the expression of molecules, such as indoleamine 2,3-dioxygenase^31^ and interleukin-17^32^. Our in vitro experiments showed that both ADSCs and ADSCs-ant expressed the gene encoding the antimicrobial peptide cathelicidin at similar levels. This implied that the combination with an antibiotic did not suppress the expression of antimicrobial peptides.

Antimicrobial peptides are evolutionarily conserved small effector molecules (10–150 amino acids) found in organisms ranging from prokaryotes to humans^33^. Antimicrobial peptide-mediated cell killing occurs by disrupting membrane integrity, by inhibiting protein, DNA, or RNA synthesis, and by interacting with certain intracellular targets^34^. Importantly, antimicrobial peptides can be active against certain pathogens that are resistant to conventional antibiotics, such as multidrug-resistant bacteria^20^. Previous studies in mice reported that cathelicidin is one of the factors produced by systemic MSCs that significantly contributes to *Staphylococcus* killing^9^. Thus, ADSCs seem to express antimicrobial peptides. This is likely, at least in part, responsible for the reduction in bacterial burden on the implant.

The capacity of MSCs to interact with the innate and adaptive immune responses to inhibit T-cell proliferation and upregulate regulatory T cells^35,36^ makes this cell population a strong candidate for cell therapy in graft-versus-host disease or vascularized composite allotransplantation. MSCs have been shown to exert immunomodulatory effects through cell contact and paracrine effects^37,38^. This protective role of MSCs in the host reportedly is dual: on one hand, they can create an immunosuppressive environment, thus avoiding exacerbation of pathological symptoms, helping to heal tissue damage, and allowing the establishment of an immune-tolerant environment; on the other hand, however, excessive immune suppression as well as the sensitivity of MSCs to microbial infection can lead to the opposite effect, hampering the host’s ability to fight the infection and, instead, encouraging the spread of microbial effectors^9^. Therefore, the immunomodulatory capacity of locally administered MSCs in infectious diseases is not fully understood. A previous study reported negative effects of MSCs on orthopaedic implant-associated bone infection^39^, which is in contrast to studies reporting a beneficial effect of intravenously administered MSCs on the development of sepsis through a reduction in systemic inflammation and increased bacterial killing and phagocytosis^39^. The authors reasoned that, although immune suppression may be beneficial in systemic infection with whole-body inflammation, local and chronic infections such as osteomyelitis may be promoted by a local immunosuppressive environment^39^. Although their infection model—consisting of a bone defect contaminated with *S.* *aureus* and administration of bone marrow-derived MSCs—was different from ours, our study revealed no negative effect of ADSCs on implant-related infection, and, in contrast, showed a positive effect of ADSCs-ant. Therefore, we conclude from the combined findings that locally injected MSCs may have an immunosuppressive capacity, but do not always promote an immunosuppressive environment, and ADSCs combined with an antibiotic are an effective option for local treatment.

Systemic ADSC-assisted antibiotics therapy offered an additional benefit by reducing acute urogenital organ damage in a rat model^40^, and ADSC therapy improved ischemia reperfusion injury not only by suppressing the inflammatory and immune responses, but also by enhancing paracrine effects^40,41^. A previous in-vitro study showed that BMSCs can uptake antibiotics^12^. Our results suggest that ADSCs can also uptake antibiotics, with the antibiotic concentration increasing over time. Whether CPFX was internalized in the cells or attached to the cell surface was not clarified in this study. However, a previous in-vitro study using confocal microscopy showed that the anti-cancer drug paclitaxel was internalized in MSCs via Golgi-derived vesicles^42^. Based on this finding, we considered that CPFX might be internalized in the ADSCs and subsequently released from the cells at the infection site. In situ drug injection probably has lower efficacy than drug-loaded MSCs because of rapid dilution of the drug. MSCs have the ability to migrate into inflammatory sites^4,43^. In vivo, MSCs have been observed to accumulate in the spleen as well as in wound areas following intravenous administration^4^. Therefore, ADSCs loaded and combined with antibiotics may improve the delivery of antibiotics to the infected area. While our results showed that ADSCs-ant had the strongest therapeutic effect in rats with implant-related infection, the additive or synergistic interaction between ADSCs and antibiotics was not elucidated in this study. Further studies are needed to determine whether there is synergistic interaction between ADSCs and CPFX, and what the optimum antibiotic, dose, and regimen are.

Systemic antibiotics alone cannot completely remove biofilms, and thus, surgical debridement is generally necessary for the treatment of implant-related infection. However, surgical debridement and revision implant have not always been successful. Achieving a high local antibiotic concentration around an infected implant is of major clinical importance, because bacteria protected by the biofilm require antibiotic concentrations that are orders of magnitude greater than the MIC required for killing the bacteria^44–46^, and an intravenous antibiotic injection is not suitable to this end^47^. Therefore, recently, direct local antibiotic injection has been highlighted as an option because it achieves high local antibiotic concentrations^47,48^. Furthermore, a recent study has shown that MSCs secrete cysteine proteases that destabilize methicillin-resistant *S.* *aureus* biofilms, thereby increasing the efficacy of antibiotics that were previously tolerated by biofilms^49^. Therefore, by using ADSCs or ADSCs-ant, the effect of local antibiotic treatment could be enhanced. However, systemic treatment is also useful as it is easier to be carried out as compared to the ease of performing a local treatment. Therefore, we are currently researching the effect of systemic ADSC treatment for implant-related infection.

The relevance of our findings to human subjects remains to be studied. In future, it will be necessary to confirm the effect of ADSCs on implant-related infection in larger animal models before clinical studies in humans can be conducted. For clinical application, the source of ADSCs is important. In our study, ADSCs were collected from allogenic rats. Autologous ADSC applications have some potential limitations. It is difficult to obtain sufficient quantities of healthy autologous ADSCs with high activity from patients with the targeted diseases^50^. Allogeneic MSCs have been previously safely administered to humans for a number of conditions, and their use as a treatment for chronic infections would not pose a unique risk^50^. One limitation of using ADSCs as a delivery vehicle for antibiotics is that their ability to do so is dependent on cell viability and integration at the injection site. By using DiI staining of ADSCs, a previous study showed that numerous ADSCs were distributed throughout granulation tissue up to 21 days post-transplantation^13^. We did not study cell viability and distribution after injection, which requires further study. Furthermore, in this in vivo study, the effect of antibiotic-loaded ADSCs alone was not assessed, but only loaded ADSCs combined with antibiotic were assessed. Therefore, the combinatorial effect of CPFX and ADSCs was shown, but the individual effect of ADSCs loaded with CPFX was not considered in this study protocol, since CPFX leaked out into the media before the cells could be administered. In the in vitro study, the concentration of CPFX in cells was higher than the MIC for *S.* *aureus*, but was substantially lower than the concentration of CPFX in the antibiotic group (100 mg/L). Therefore, we expected not only antibiotic delivery in cells, but also a synergistic or additive effect of the administered antibiotic and loaded ADSCs.

In summary, ADSCs can uptake antibiotics without suppression of antimicrobial peptide gene expression. Injected ADSCs exerted an antimicrobial effect, and local administration of ADSCs with CPFX suppressed chronic *S.* *aureus* infection in implant-related osteomyelitis. These findings suggest that local ADSC therapy combined with an antibiotic represents a novel treatment strategy for patients with implant-associated osteomyelitis. The results of this study highlight the potential use of this combined regimen in patients with implant-related osteomyelitis who responded poorly to conventional medical treatment.

## Methods

ADSC and BMSCs for in vitro experiments were isolated from 20 9-week-old female Wistar rats (Japan SLC Co., Shizuoka, Japan). ADSCs were prepared as previously reported^13^, with modification. BMSCs were isolated from the same rats, as previously reported^51^, with modifications in the protocol. Further details can be found in the Supplementary information.

### Assay of sensitivity of ADSCs to CPFX

The anti-proliferative and cytotoxic effects of CPFX (Wako) on rat ADSCs were determined by a 3-(4,5-dimethyl-2-thiazolyl)-2,5-diphenyl-2-H-tetrazolium (MTT) assay (Sigma-Aldrich, St. Louis, MO, USA) as previously reported^12^, with modification. CPFX is a fluoroquinolone and is considered a drug of choice for the treatment of osteomyelitis because it penetrates into poorly vascularized sites of infection^12^. Cells were seeded in 96-multiwell plates at 10,000 cells/well in 100 μL of Dulbecco’s modified Eagle medium (DMEM) per well. In the anti-proliferative assay, cells were incubated for 24 h or 7 days with various concentrations of CPFX (1.95–250 mg/L, with 1:2 dilutions from one concentration to the following one). At the end of incubation, cell proliferation or viability was evaluated by MTT assay.

### CPFX loading of ADSCs and BMSCs

ADSCs and BMSCs (1 × 10^5 ^cells/mL) were plated in 100-mm dishes containing DMEM including fetal bovine serum (FBS) and CPFX (100 mg/L) for 10 min, 1 h, 12 h, or 24 h, as previously described^12^. At the end of the incubation, the cells were washed three times with phosphate-buffered saline (PBS). After loading with CPFX, the cell medium was changed to DMEM without CPFX. The concentration of CPFX released from ADSCs and BMSCs after the medium exchange was measured in 1 mL of medium obtained at 24, 48, or 72 h. The medium was exchanged each time after sample collection.

### Assay of the ability of ADSCs and ADSCs-ant to differentiate into osteoblasts

Antibiotic-loaded ADSCs and ADSCs were analysed for their capacity for osteogenic differentiation using ALP staining and alizarin red histochemistry. To induce differentiation, cells were cultured in osteogenic medium composed of α-MEM (Wako Pure Chemical Industries) containing 10% FBS, 0.1 mM dexamethasone, 50 mM ascorbate-2-phosphate, 10 mM β-glycerophosphate, and 1% penicillin-streptomycin^52^. Alkaline phosphatase (ALP) histochemistry was performed at 2 weeks after osteogenic induction culture. For ALP staining, cells were rinsed with PBS three times and fixed in 4% paraformaldehyde phosphate buffer (Wako) at room temperature for 5 min. They were then washed with deionized water. The fixed cells were incubated with 1-Step NBT/BCIP plus Suppressor Solution (Thermo Fisher Scientific) at 37 °C for 30 min, washed with deionized water, and observed both with the naked eye and under a light microscope (Biorevo BZ-9000; Keyence, Osaka, Japan). For alizarin red staining, cells were rinsed with PBS three times, fixed in 4% paraformaldehyde phosphate buffer, and stained using an Osteogenesis Assay Kit (ECM815; Millipore) per the manufacturer’s instructions.

### RT-qPCR

The mRNA expression of rat *osteocalcin*, rat *ALP*, and rat *CRAMP* was evaluated by qPCR. Briefly, RNA was extracted from the cells, and cDNA was generated using RNA to cDNA EcoDry Premix (Oligo dT) (Takara Bio, Kusatsu, Japan). qPCRs were run using THUNDERBIRD SYBR qPCR Mix (Toyobo, Tokyo, Japan) and the following primer sets: 5′-GACTGCATTCTGCCTCTCTG-3′ and 5′-ATTCACCACCTTACTGCCCT-3′ for *osteocalcin*, 5′-AACAACCTGACTGACCCTTC-3′ and 5′-TCCACTAGCAAGAAGAAGCC-3′ for *ALP*, 5′-GGTTCCGAGTGAAGGAGACTG-3′ and 5′-TACCAGGCGCATCACAACTG-3′ for *rCRAMP*, and 5′- ATCACCATCTTCCAGGAGCG-3′ and 5′-CCTTCTCCATGGTGGTGAAG-3′ for rat glyceraldehyde-3-phosphate dehydrogenase (*Gapdh*)^53,54^. Target mRNA levels were normalized to that of *Gapdh*.

### Liquid chromatography-tandem mass spectrometry (LC–MS/MS) for the measurement of the concentration of CPFX in ADSCs and BMSCs, and CM

The concentrations of CPFX in ADSCs and CM, and in BMSCs and CM as a control, were quantified using LC–MS/MS, as previously reported^12^. CPFX concentrations in cells were determined at 10 min, 1 h, 12 h, and 24 h after treatment with CPFX (100 mg/L) as described above, and those in CM at 24, 48, and 72 h after medium exchange. Furthermore, CPFX concentrations in cells after release were analysed at 24, 48, and 72 h. To assess potential adsorption of CPFX to the plate, we measured the concentration of CPFX in DMEM without cells after the changing the medium following a 24-h incubation with DMEM containing 100 mg/L CPFX, without cells. Detailed methods are described in the Supplementary information.

### Assay of the antimicrobial activity of cells and CM of ADSCs-ant

The concentration of CPFX in antibiotic-loaded ADSCs was assessed using the broth microdilution method in cation-adjusted Mueller–Hinton broth^55^. Antibiotic-loaded ADSCs and the CM of the antibiotic-loaded ADSCs were tested for their activity on *S.* *aureus* strain ATCC29213 (American Type Culture Collection, Manassas, VA, USA). Further details of the method can be found in the Supplementary information.

### In-vivo study

**Figure 7:**
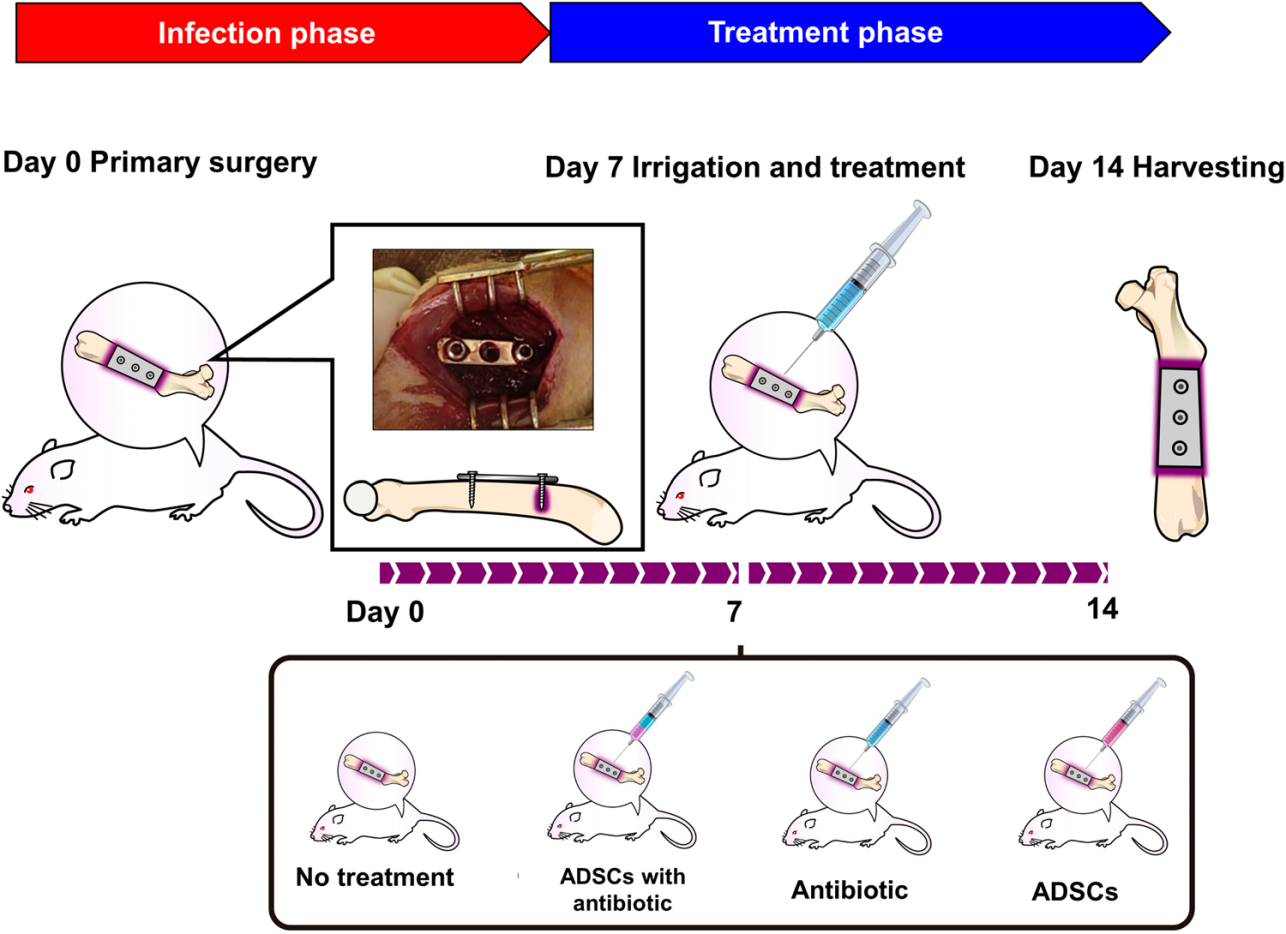
Protocol for implant-related infection model establishment in rats.

The protocol for establishing the implant-related infection model is shown in Fig. 7. Thirty-six 9-week-old female Wistar rats were purchased from Japan SLC Co. (Shizuoka, Japan). Twelve rats were used to obtain ADSCs and the remaining 24 rats were randomly assigned to four treatment groups. Prior to surgery, the rats were sedated and anesthetized with medetomidine (0.5 mg/kg) (Zenoaq, Fukushima, Japan), midazolam (2.5 mg/kg) (Astellas Pharma, Tokyo, Japan), and butorphanol tartrate (2.5 mg/kg) (Meiji Seika Pharma, Tokyo, Japan) injected intraperitoneally. Animals were allowed full activity in their cages and were medicated with meloxicam (0.2 mg/kg) on post-operative day two. Their drinking and feeding behaviours were monitored regularly. The left femur was exposed by a direct lateral approach, and a 3-hole stainless plate was fixed to the femur with two stainless screws inserted into the outermost screw holes of the plate (straight miniplate, DePuy Synthes, Raynham, MA, USA). To establish infection, the distal screw was incubated in an overnight culture of *S.* *aureus* strain ATCC29213 and air-dried for 20 min prior to insertion using a previously reported method^56^. The wound was then closed with nylon sutures. Seven days after the primary surgery, the rats were sedated and anesthetized again, and the surgical scar was reopened and irrigated with 10 mL of PBS. Rats were clarified for 4 groups: rats in the no-treatment group (n = 6) were injected using an 18-gauge needle locally into the surgical site with DMEM (2 mL), rats in the ADSCs-ant group (n = 6) with ADSCs-ant (1 × 10^5^ ADSCs/mL pre-loaded with CPFX in DMEM containing CPFX 50 mg/L, 2 mL), and rats in the in antibiotic group (n = 6) with CPFX alone (2 mL of DMEM containing 100 mg/L CPFX), and rats in the ADSCs group (n = 6) with ADSCs alone (2 mL of DMEM containing 1 × 10^5^ ADSCs/mL). In the ADSCs-ant group, ADSCs loaded with CPFX were washed, harvested by trypsinization with 0.05% trypsin, and resuspended in DMEM containing 100 mg/L CPFX for preservation until use so that the final CPFX concentration was 50 mg/L. The ADSCs, ADSCs-ant, and DMEM with CPFX were produced in the laboratory at the Department of Orthopaedic Surgery, Kanazawa University Graduate School of Medical Sciences, and then transferred to the laboratory at the Institute for Gene Research, Kanazawa University immediately. The rats were euthanized on day 7 post injection (14 days after infection) after evaluating the general impression and soft tissue swelling. After euthanization, abscess formation was evaluated to reopen the surgical scar.

### Ex vivo analyses

Rats were euthanized on day 14 post primary surgery (day 7 post injection), and the implants and femurs were harvested in a sterile manner for ex-vivo analyses. Osteomyelitis was scored by two examiners (Y.J. and Y.Y.) according to a modified score reported previously^39,57,58^ (Supplementary Table 1).

### Modified osteomyelitis scores

Modified osteomyelitis scoring by two examiners was based on (1) general impression, (2) soft tissue swelling, (3) abscess formation, (4) proximal screw loosening, and (5) distal screw loosening. In case of disagreements between the two examiners, the lowest score was taken. Parameters 1–3 ranged from 0 (good or absent), 1 (mild), 2 (moderate) to 3 (bad or severe). Parameters 4 and 5 were judged based on micro-CT images, which was as follows: We calculated the degree of osteolysis as a healthy bone ratio (cortical bone area/total bone area). A mean ratio of > 0.7 was scored as 0, 0.6–0.7 was scored as 1, < 0.6 was scored as 2, and fracture was scored as 3. The maximum score to be achieved was 15 [5 parameters, 3 points maximum score (see Supplementary Table 1)].

### μCT analysis

At post-surgery day 14 (day 7 post revision), the plated femurs were disarticulated, the implant and soft tissue were removed carefully, and the samples were subjected to μCT scanning at 10.5-micron resolution (LaTheta LCT-200; Hitachi-Aloka, Tokyo, Japan). To quantify osteolysis in the screw holes, μCT images in digital imaging and communications in medicine (DICOM) format were obtained for volumetric osteolysis analysis using the DICOM viewer software Synapse Vincent (Version 5; Fujifilm, Tokyo, Japan, https://www.fujifilm.com/jp/ja/healthcare/healthcare-it/it-3d/vincent#). The femoral bone was automatically traced as the region of interest, and the Hounsfield Unit (HU) value was calculated^59^. The osteolytic volume of the screw hole was determined by calculating the total screw hole volume of the 5 slices and comparing the cortical bone (voxels ≥ 230 Hu) within the total screw hole volume^56^. We calculated the degree of osteolysis as the healthy bone ratio (cortical bone area/total bone area).

### Histological analysis

The femoral bone was fixed in 10% neutralized formalin solution and dehydrated using an ethanol gradient (70%, 80%, 90%, and 100%). The fixed specimens were decalcified in 10% formic sodium citrate solution, embedded in paraffin, and sectioned in the coronal plane at 0.2-μm thickness. The sections were stained with haematoxylin and eosin, and the slides were observed under an optical microscope (Biorevo BZ-9000; Keyence, Osaka, Japan). The abscessed area in the total area was evaluated in three regions, including at the distal screw hall, the proximal screw hall, and the region between the two screw halls. The assessment was confirmed by a pathologist (N.T.).

### CFU assay

The bacterial burden on the implants was determined by CFU assay following sonication, as previously described^60^. Briefly, the implants were placed into 1 ml PBS in 1.5 ml microtubes. The solution was subjected to rapid vortex mixing for 15 s and then sonicated for 5 min (Bransonic Branson 5,210, Kanagawa, Japan) at a frequency of 40 Hz to disrupt the formed biofilm. Finally, rapid vortex mixing of the solution was performed again for 1 min. This method of disrupting the biofilm was performed in accordance with the method reported by Braem et al.^61^, with slight modification. CFU assays were performed on the explanted proximal (sterile) screws, distal (contaminated) screws, and plates and soft tissues around the implant obtained on day 14 after surgery.

### Statistical analysis

Data are reported as the median ± interquartile range. The Shapiro–Wilk test was used to check normal distribution, and the Bartlett’s test was used to evaluate equality of variances. Means of two groups were compared using unpaired Student’s *t*-tests. Multiple groups were compared using one of the following: ordinary one-way ANOVA followed by Sidak’s post-hoc test (for normally distributed data with equal variances), Brown-Forsythe and Welch ANOVA followed by Dunnett’s T3 post-hoc test (for normally distributed data without equal variances), or Kruskal–Wallis test followed by Dunn’s post-hoc test (for non-normally distributed data). *P* < 0.05 was considered significant. All analyses were conducted using Prism8 software (Version 8.1.2.332; GraphPad Software, San Diego, CA, https://www.graphpad.com/scientific-software/prism/).

### Ethics approval

The investigational protocol was approved by the Kanazawa University Advanced Science Research Centre (Approval Number: AP-173878), and all animals were treated in accordance with Kanazawa University Animal Experimentation Regulations.

## Supplementary information

Supplementary information

## Data Availability

All the data used to draw the conclusions of this paper are available in the data presented in the figures and/or table. The raw/processed data required to reproduce these findings are available from the corresponding author upon request.
