# Supplementary material for: Combinational therapy with antibiotics and antibiotic-loaded adipose-derived stem cells reduce abscess formation in implant-related infection in rats
Source: Sci Rep. 2020 Jul 7;10:11182. doi: 10.1038/s41598-020-68184-y (PMC7341734; doi:10.1038/s41598-020-68184-y)
Supplement: Supplementary file 1 — Supplementary information [file 41598_2020_68184_MOESM1_ESM.docx]

**Supplementary Information**

**Combinational therapy with antibiotics and antibiotic-loaded adipose-derived stem cells reduce abscess formation in implant-related infection in rats**

Junya Yoshitani, Tamon Kabata, Hiroshi Arakawa, Yukio Kato, Takayuki Nojima, Katsuhiro Hayashi, Masaharu Tokoro, Naotoshi Sugimoto, Yoshitomo Kajino, Daisuke Inoue, Ken Ueoka, Yuki Yamamuro & Hiroyuki Tsuchiya

**Supplementary Methods**

**Details of preparation of ADSCs and BMSCs**

ADSCs were prepared as previously reported^1^, with modification. Briefly, adipose tissue (1.5 g) was harvested from the right inguinal region of a rat and washed with phosphate-buffered saline (PBS; Wako, Osaka, Japan). The tissue was cut into strips within 5 min. Collagenase (Wako) was dissolved in 20 mL of PBS at a final concentration of 0.12% and was used to digest adipose tissue in a water bath at 37°C for 45 min. The collagenase activity was neutralized by the addition of 20 mL of Dulbecco’s modified Eagle’s medium (DMEM; Wako) containing 10% foetal bovine serum (FBS; Nichirei Bioscience), 100 U/mL penicillin, and 100 mg/mL streptomycin (P/S; Wako). Then, the solution was filtered and collected in a tube and centrifuged at 1,500 rpm at 25°C for 5 min, and the supernatant was discarded. The pelleted ADSCs were resuspended in 10 mL of DMEM containing 10% FBS and P/S and plated into 15-cm-diameter culture dishes and cultured at 37°C in the presence of 5% CO_2_ for one week.

BMSCs were isolated as previously reported^2^, with modification. A rat was sacrificed, the pelt was wetted thoroughly with 70% isopropanol, and the hind limbs were clipped and peeled. The knee joint was cut in the centre using sterile sharp scissors, and the ligaments and excess tissue were removed. At the same time, the femur was severed at the hip. The surrounding muscles, ligaments, and excess tissue were detached from the bone. The ends of the long bones were trimmed to expose the interior marrow shaft. Both femoral epiphyses were cut and the medulla was carefully flushed with 3 mL of L‑DMEM containing 10% FBS and P/S using a syringe with an 18‑gauge needle. Using the same needle and syringe, on ice, medium and cells were gently drawn up and down several times to produce a single‑cell suspension. The bone-marrow suspensions were cultured in polystyrene 100-mm dishes, and non‑adherent cells were removed from the culture after two days by a series of washes in PBS and subsequent changes of medium. Adherent cells were expanded as monolayer cultures in a 5% CO_2_/95% air atmosphere at 37°C. The culture medium of ADSCs and BMSCs was changed every two days to remove nonadherent cells. When the cells reached 80–90% confluence (1 × 10^5^ cells/mL), they were detached with 0.05% trypsin for subculture.

**Liquid chromatography-tandem mass spectrometry (LC-MS/MS) for the measurement of the concentration of CPFX in ADSCs and BMSCs and conditioned media (CM)**

For CM samples, 5 μL of medium was diluted with 245 μL of 0.1% formic acid in water and 250 μL of acetonitrile containing 0.1% formic acid and 50 ng/mL levofloxacin as an internal standard. After mixing, the samples were immediately centrifuged at 21,500 × *g* at 4°C for 5 min, and the supernatants were subjected to LC-MS/MS. For cell samples, cells were collected in 1 mL of 0.25% trypsin solution and added to 9 mL of culture medium. Suspended cells (100 μL) were diluted in 150 μL of 0.1% formic acid in water and 250 μL of acetonitrile containing 0.1% formic acid and 50 ng/mL levofloxacin. After mixing, the samples were centrifuged at 21,500 × *g* at 4°C for 5 min, and the supernatants were subjected to LC-MS/MS.

The amounts of CPFX were determined with a LCMS-8050 triple quadrupole mass spectrometer (Shimadzu, Kyoto, Japan) coupled to an LC-30A system (Shimadzu) using a C18 ODS MGIII (3 μm 2.0 mm I.D. × 50 mm, Osaka Soda, Osaka, Japan) at 40°C. The mobile phase was composed of a mixture of 0.1% formic acid in water (pH 3.0) and 0.1% formic acid in acetonitrile, and the flow rate was 0.4 mL/min. The gradient program was as follows: 10% B for 30 s, 10–35% B for 18 s, 35% B for 42 s, 35–80% B for 60 s, 80% B for 120 s, 80–10% B for 30 s, and 10% B for 90 s. The mass numbers of the molecular and product ions for each compound were as follows: ciprofloxacin (332.15→231.15, CE –36 V), levofloxacin (362.2→261.2, CE –27 V). LabSolutions software (version 5.89; Shimadzu, https://www.an.shimadzu.co.jp/labsolutions-cs/index.htm) was used for data manipulation. The detection limit was 1 ng/mL for each compound.

**Anti-microbial activity assay of cells and CM of ADSCs-ant**^3^

Twofold serial dilutions of cells and CM were prepared in a 96-well microtiter plate containing 100 μL of cation-supplemented Mueller-Hinton broth per well. *S. aureus* inocula were adjusted to 0.5 McFarland (equivalent to 1 × 10^8^ colony-forming units (CFU)/mL). The minimum inhibitory concentration (MIC) was determined as the lowest concentration that completely inhibited growth. To exclude any specific effects, ADSCs, CM of ADSCs, and CPFX were also tested as controls. MICs were determined by visual inspection of cell growth in each well. All MIC determinations were performed in duplicate to ensure reproducibility of the results.

**References**

1. Sisto, F. *et al*. Human mesenchymal stromal cells can uptake and release ciprofloxacin, acquiring *in vitro* anti-bacterial activity. *Cytotherapy* **16**, 181–190 (2014).

2. Song, K., Huang, M., Shi, Q., Du, T. & Cao, Y. Cultivation and identification of rat bone marrow-derived mesenchymal stem cells. *Mol. Med. Rep.* 10, 755–760 (2014).

3. Jorgensen, J.H. & Ferraro, M.J. Antimicrobial susceptibility testing: a review of general principles and contemporary practices. *Clin. Infect. Dis.* **49**, 1749–1755 (2009).

**Supplementary Table 1. Modified osteomyelitis scores**

| Parameter | | Score | | | |
| --- | --- | --- | --- | --- | --- |
|  |  | 0 | 1 | 2 | 3 |
| 1 | General impression | good | Mild | moderate | Bad |
| 2 | Soft tissue swelling | absent | Mild | moderate | Severe |
| 3 | Abscess formation | absent | Mild | moderate | Severe |
| 4 | Screw loosening proximal | 0.7 < mean ratio | 0.6 < mean ratio < 0.7 | 0.6 > mean ratio | Fracture |
| 5 | Screw loosening distal | 0.7 < mean ratio | 0.6 < mean ratio < 0.7 | 0.6 > mean ratio | fracture |

HU, Hounsfield units


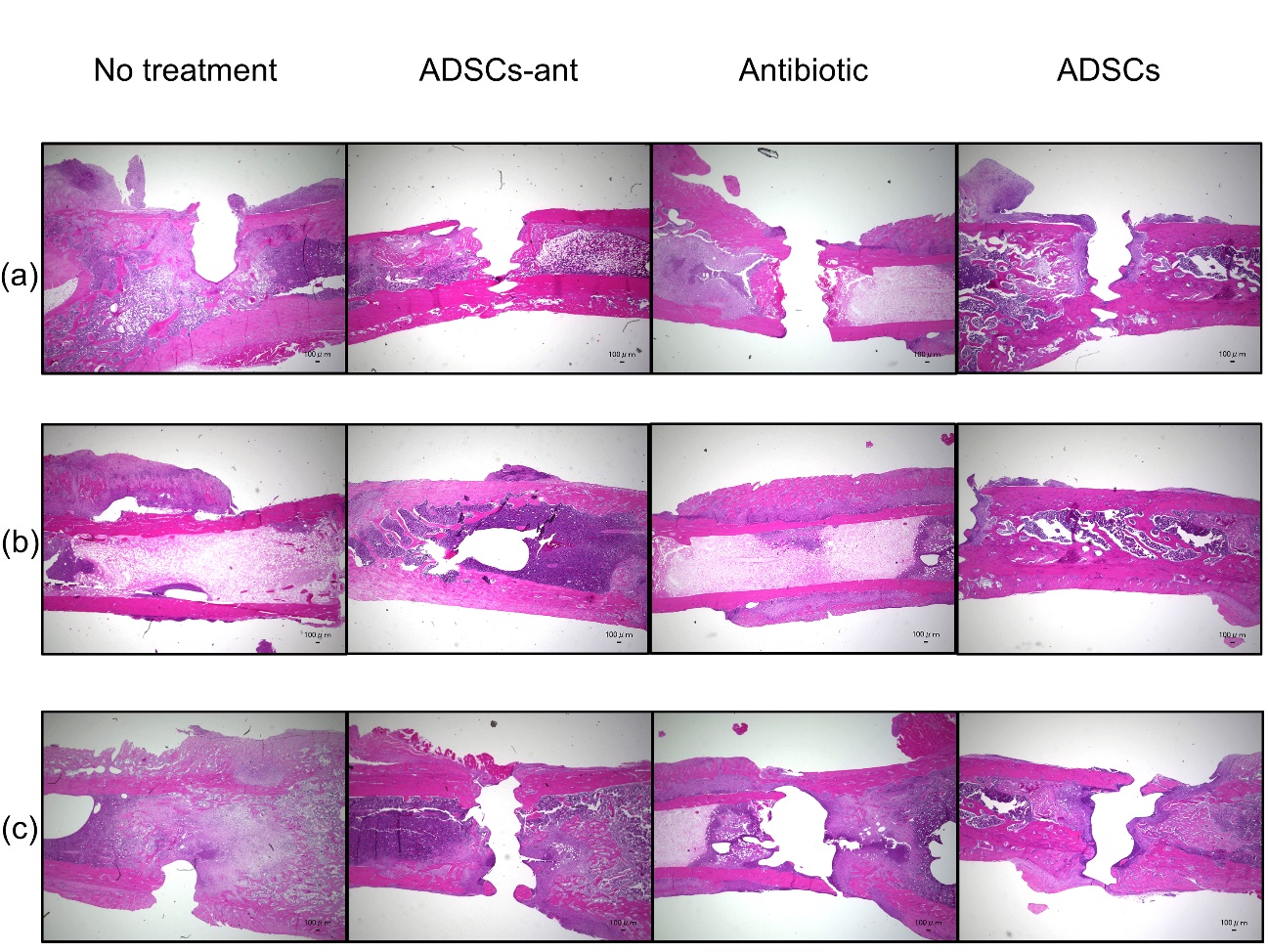


**Supplementary Fig. S1** Histology at the proximal screw hall (a), middle area (b), and distal screw hall (c) in all treatment groups.
